# Sigmoid volvulus: Comorbidity with sigmoid gangrene

**DOI:** 10.12669/pjms.35.1.295

**Published:** 2019

**Authors:** Sabri Selcuk Atamanalp, Esra Disci, Refik Selim Atamanalp

**Affiliations:** 1 Prof. Sabri Selcuk Atamanalp, MD. Department of General Surgery, Faculty of Medicine, Ataturk University, Erzurum, Turkey; 2 Esra Disci, MD. Assistant Professor, Department of General Surgery, Faculty of Medicine, Ataturk University, Erzurum, Turkey; 3 Refik Selim Atamanalp, MD. Assistant Professor, Department of General Surgery, Faculty of Medicine, Ataturk University, Erzurum, Turkey

**Keywords:** Sigmoid Colon, Volvulus, Sigmoid Gangrene, Endoscopy, Surgery

## Abstract

Sigmoid volvulus (SV) is the wrapping of the sigmoid colon around its mesentery, and sigmoid gangrene is a catastrophic complication of SV. Although the diagnosis of SV is generally not difficult, unfortunately, most of the clinical, laboratory and radiological signs are not pathognomonic in demonstrating sigmoid gangrene. The treatment of gangrenous SV requires emergency surgery. Sigmoid gangrene worsens the prognosis of SV by doubling the mortality rate.

## INTRODUCTION

Sigmoid gangrene is seen in 6.1-93.4% of cases with sigmoid volvulus (SV).^1^,^2^ Although some clinical and laboratory findings such as melanotic stool, fever, leucocytosis, abdominal guarding/rebound tenderness, hypotension/shock, somnolence and metabolic acidosis suggest the sigmoid gangrene, most of them generally fail in accurate diagnosis.^3^,^4^ Similarly, although some radiological studies including Doppler ultrasonography, angiography or scintigraphy demonstrate the vascular occlusion, they are generally inadequate in determining sigmoid gangrene.^5^ When considerations are suitable, endoscopy identifies mucosal viability.^6^ Emergency surgery is needed to treat gangrenous SV, and primary anastomosis or stoma is used together with the resection of the gangrenous sigmoid colon.^7^,^8^

Despite SV being rare worldwide,^3^ it is relatively common in Eastern Anatolia,^9^ where we live. Our clinic has approximately 52 years of history and 1,008 cases of experience with SV, which is the largest single-centre SV series over the world.^10^ We wanted to utilize the present data and experience to evaluate the comorbidity of SV with sigmoid gangrene.

## CLINICAL EXPERIENCE

**Fig.1:**
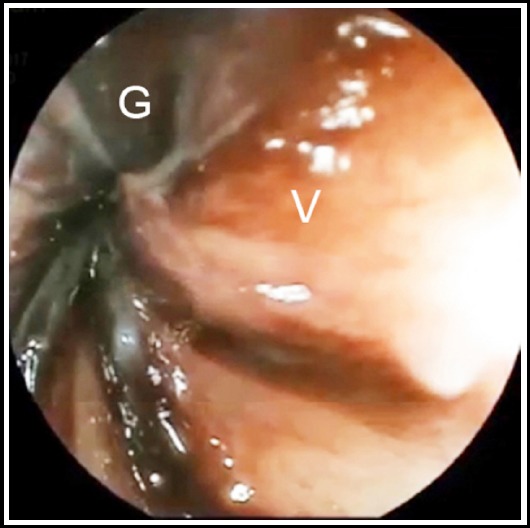
Endoscopic appearance of sigmoid gangrene in sigmoid volvulus (G: gangrenous mucosa, V: viable mucosa).

**Fig.2:**
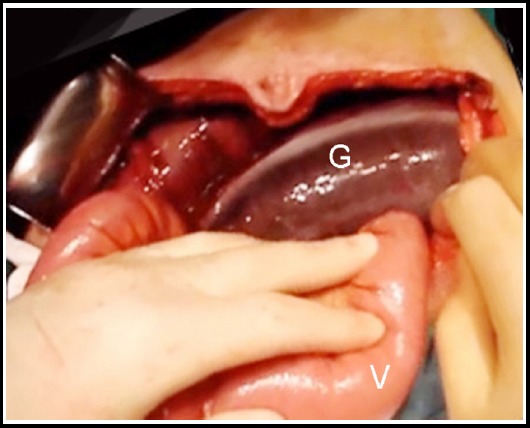
Operative appearance of sigmoid gangrene in sigmoid volvulus (G: gangrenous sigmoid colon, V: viable small intestine)

In our 1,008-case SV series, sigmoid gangrene was determined in a total of 284 patients (28.2%). Diagnosis was made by digital rectal examination in 96 cases, by endoscopy (Fig.1) in 44, and by laparotomy (Fig.2) in the remaining. In our experiments, sigmoid gangrene is generally limited to the twisted segment or a few cm. from the proximal and distal lines, but it rarely extends to the descending colon or rectum, only occurring in patients with very late admission, over-rotation or ileosigmoid knotting.

## DISCUSSION

In SV, sigmoid gangrene is seen in 6.1-30.2% of all patients and in 10.7-93.4% of surgically treated cases.^1^,^2^ Well-known factors affecting bowel gangrene development are the presence of major comorbidities (e.g., cardio-vascular diseases), shock (hypovolemic and/or toxic), delayed admission to the hospital, over-rotation (twisting degree>360°), and ileosigmoid knotting.^2^,^3^,^11^,^12^

In SV, clinical presentation originally arises from the precursor pathology, acute closed-loop intestinal obstruction. Additionally, the bloodstream is decreased or interrupted by the twisting of the mesentery. If the process continues for a few hours, the secondary pathology, bowel ischaemia and gangrene, and related clinical features are supervened.^4^,^13^ In addition to the initial hypovolemia, due to volume loss into the obstructive bowel lumen, necrosis facilitates bacterial translocation and the absorption of toxic products, resulting in toxaemia.^2^

The prominent clinical and laboratory indicators of sigmoid gangrene are melanotic stool, fever, leucocytosis, abdominal guarding/rebound tenderness, hypotension/shock, somnolence and metabolic acidosis. Except for the melanotic stool, these indicators are not pathognomonic.^3^,^4^ Doppler ultrasonography, angiography or scintigraphy may demonstrate the vascular occlusion, but this is not a mystery.^5^ If there is no contraindication, sigmoidoscopy or colonoscopy is a unique method to identify the mucosal viability.^6^ Endoscopic signs of bowel gangrene include devitalized brown-black mucosa and gangrenous blood-stained effluent.^5^,^6^ In surgery, bowel gangrene is easily diagnosed by means of a brown-black bowel segment.^8^

In SV, when bowel gangrene is determined or suspected in the preoperational period, emergency surgery is required without trying an endoscopy. If gangrene is diagnosed in an endoscopy, the procedure is terminated, and emergency surgery is planned. In operation, following the resection of the gangrenous segments, primary anastomosis is preferred in non-elderly and well-conditioned patients, while stoma may be life-saving in some elderly or bed-conditioned cases.^7^,^8^,^13^,^14^ Bowel gangrene worsens the prognosis by increasing the mortality rate from 0-40% to 3.7-80%.^2^,^9^

## CONCLUSION

Sigmoid gangrene is a life-threating complication of SV. Even if the prognosis is generally poor, the observation of the basic diagnostic indicators and following the above mentioned basic therapeutic rules can relatively reduce the prognosis.

### Authors’ Contribution

**SSA:** designed the study, collected and analysed the data, reviewed the literature and prepared the manuscript.

**ED:** Collected the data, RSA Prepared the manuscript.
